# Sex Differences in Familial Hypercholesterolemia

**DOI:** 10.1007/s11883-023-01155-6

**Published:** 2023-10-10

**Authors:** Marianne Klevmoen, Janneke W.C.M. Mulder, Jeanine E. Roeters van Lennep, Kirsten B. Holven

**Affiliations:** 1 Department of Nutrition, Institute of Basic Medical Sciences, University of Oslo, Oslo, Norway; 2 Norwegian National Advisory Unit on Familial Hypercholesterolemia, Oslo University Hospital, Oslo, Norway; 3 Department of Internal Medicine, Cardiovascular Institute, Erasmus MC Cardiovascular Institute, University Medical Center Rotterdam, Rotterdam, Netherlands

**Keywords:** Familial hypercholesterolemia, Cardiovascular disease, Cholesterol, Sex differences, Pregnancy, Breastfeeding

## Abstract

**Purpose of Review:**

This review aims to summarize the existing research on sex differences in familial hypercholesterolemia (FH) across the lifespan.

**Recent Findings:**

From childhood onward, total- and low-density lipoprotein cholesterol (LDL-C) levels in girls are higher than those in boys with FH. By the age of 30 years, women with FH have a higher LDL-C burden than men. In adulthood, women are diagnosed later than men, receive less lipid-lowering treatment, and consequently have higher LDL-C levels. An excessive atherosclerotic cardiovascular disease risk is reported in young female compared to male FH patients. The periods of pregnancy and breastfeeding contribute to treatment loss and increased cholesterol burden.

**Summary:**

Earlier initiation of treatment, especially in girls with FH, and lifelong treatment during all life stages are important. Future research should aim to recruit both women and men, report sex-specific data, and investigate the impact of the female life course on cardiovascular outcomes. Future guidelines should include sex-specific aspects.

**Graphical abstract:**

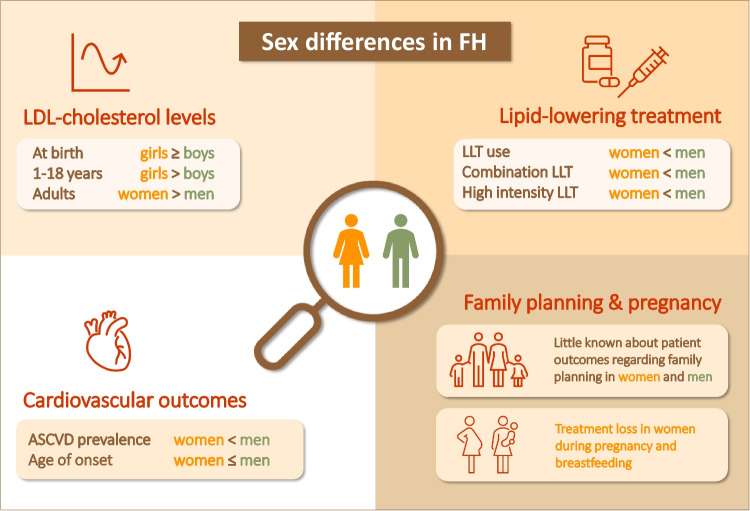

## Introduction

Familial hypercholesterolemia (FH) is an autosomal dominant disease, causing elevated plasma levels of low-density lipoprotein cholesterol (LDL-C) from childhood onward [1]. LDL-C is a causal risk factor for atherosclerotic cardiovascular disease (ASCVD) [2], and the cholesterol burden through life defines the risk of ASCVD [3, 4]. Early and lifelong treatment is therefore crucial. FH is most commonly caused by mutations in the genes encoding low-density lipoprotein receptor (*LDLR*), apolipoprotein B (*APOB*), and proprotein convertase subtilisin/kexin type 9 (*PCSK9*) [1]. The prevalence of heterozygous FH, hereafter referred to as FH, was recently estimated in a worldwide analysis of 11 million subjects to be 1:313 in the general population, 1:31 among subjects with ischemic heart disease, and 1:15 among subjects with premature ischemic heart disease [5]. The prevalence of FH is similar in women and men. Homozygous FH (HoFH), defined by having bi-allelic pathogenic mutations in FH genes, is rare with an estimated prevalence of 1 in 250,000–360,000 [6].

Lifestyle advice and lipid-lowering drug treatment are the main approaches in treatment of FH. Lifestyle advice includes having a heart-healthy diet, sufficient physical activity, and no smoking. Lipid-lowering drug treatment is recommended from 8 to 10 years of age [7]. The cornerstone of lipid-lowering medication are statins, which are often combined with ezetimibe and if needed PCSK9 inhibitors. Several studies have shown sex differences in lipid levels and treatment in women and men with FH [8, 9•, 10•• ].

FH patients have an increased risk of premature ASCVD. More than 90 % of patients with FH experience ASCVD during their lifetime [11]. The mean age at first ASCVD event was 44 years in a Norwegian cohort study [11]. Moreover, mean age of death among FH patients is observed at 62 years, which is 15 to 21 years earlier compared to women and men from the general population [12].

In patients with FH, the age at first cardiovascular event was observed to occur at a similar age in women and men [11]. This contrasts with the general population, where the first ASCVD event occurs 7–10 years later in women than that in men [13, 14]. An excessive ASCVD risk in, mainly younger, female FH patients compared to that in male FH patients has been reported in several cohorts [15•, 16].

Awareness of sex differences in both treatment and possible patient outcomes is important. Hence, sex-specific research can ultimately lead to better patient care. The purpose of this review is therefore to summarize the currently available literature on sex differences in FH patients during the life course, diagnosis, treatment, ASCVD outcomes, and family planning periods.

## Lipids During Life Course

Levels of total cholesterol and LDL-C are low in cord blood in neonates with and without FH. However, already from the age of 1 year, the LDL-C levels reach adult levels [17]. Girls with FH have considerably higher total cholesterol and LDL-C levels than boys with FH in childhood and adolescence up to the age of 20 years [8]. An approximate mean difference of 0.5 mmol/L and 0.4 mmol/L higher total and LDL-C, respectively, has been observed in girls with FH compared to boys with FH, leading to a higher cholesterol burden among young women with FH compared to young men with FH [8]. Even though this might seem a modest difference, the lifelong effect of 0.4 mmol/L in LDL-C can be illustrated by the effect of LDL-C lowering single-nucleotide polymorphisms, where a study reported a 50 % reduction in the risk of coronary heart disease in carriers who had a mean 0.3 mmol/L lower LDL-C than controls [8, 18].

A recent study showed that at the age of 30 years, women with FH had an LDL-C burden of mean 175 mmol-years compared to 156.7 mmol-years in men with FH. In women and men without FH, the LDL-C burden was lower and similar in both sexes at age 30 years (78 and 78.6 mmol-years, respectively) [19•]. An LDL-C burden of 125 mmol-years has been suggested as a threshold for increased risk of MI, followed by a threshold of 220 mmol-years at which MI occurs [3].

In adults, women with FH have in general higher LDL-C levels than men with FH. Women have higher LDL-C levels both before treatment initiation and while receiving lipid-lowering treatment [9•, 20, 21].

Lp(a) levels in the general population are described as approximately 5–10 % higher in women than those in men and Lp(a) increases during pregnancy and at menopause in women [22, 23]. In the FH population, Lp(a) levels were similar in women and men with FH in the total study population. However, Lp(a) was higher in women with FH who were susceptible for coronary heart disease (CHD) than women who were not susceptible for CHD, highlighting the importance of taking Lp(a) into account in risk assessment especially in women [24].

In the general population, studies show that lipid levels fluctuate during different phases of the menstrual cycle and also that lipid levels substantially increase after menopause [25, 26]. In women with FH, little is known about the impact of different female life stages, such as the menstrual cycle and menopause, on lipid levels, and ASCVD outcomes. Future research should investigate these aspects.

With regard to sex differences in HoFH patients, a Canadian study in 1993 with 21 patients reported there were no sex differences in LDL-C levels [27]. More recently, the Homozygous Familial Hypercholesterolaemia International Clinical Collaboration (HICC) registry, a worldwide registry of 751 patients from 38 countries, also did not observe differences in treated or untreated LDL-C levels between women and men [28].

## Treatment

In general, women with FH are diagnosed approximately 3 to 7 years later than men [10••, 15•, 29, 30]. This leads to an increased cholesterol burden in women compared to men already early in life. In addition, once diagnosed, women with FH are less often treated with high-intensity statin [9•, 10••, 20, 29–32] or combination treatment with PCSK9 inhibitors and/or ezetimibe than men [10••, 20, 31]. A meta-analysis with individual patient data from 27 statin trials found no difference in LDL-C lowering between women and men, indicating no sex differences in LDL-C response to statins [33]. Consequently, women with FH are less likely to achieve recommended LDL-C treatment goals than men with FH [10••, 29–31].

Reasons for sex differences in treatment and goal achievement in FH are not fully known but may be multifactorial. For example, women report more side effects with statins, possibly hampering use and/or up-titration to optimal lipid-lowering treatment [9•, 29]. Another reason could be physician related. In a Spanish study containing data from 3361 FH patients, women had a 49 % lower chance of receiving a PCSK9 inhibitor prescription than men [31]. Even when women with FH are prescribed PCSK9 inhibitors, women compared to men show less LDL-C reduction of PCSK9 inhibitors [34, 35]. A reason for this sex difference may be related to the influence of estrogens on PCSK9 levels through G-protein coupled estrogen receptors on the hepatocytes, and this should be further investigated [36, 37].

Previous studies of FH cohorts report no sex differences in adherence to lipid-lowering medication [38–40]. Further investigation into possible reasons for sex differences in treatment is necessary to provide insight into how to increase the number of women with FH reaching target LDL-C levels.

In patients with HoFH, no sex differences were observed in the age at diagnosis or the type and intensity of lipid-lowering treatment [28]. In addition, treated LDL-C levels and LDL-C goal achievement was comparable between women and men with HoFH in the HICC registry.

## Cardiovascular Outcomes

In the general population, the prevalence of ASCVD events is lower in women than in men [14]. In the FH population, studies show no sex differences in the prevalence of ASCVD events [32, 41, 42]. However, some studies report lower prevalence of ASCVD in women than that in men also with FH [10••, 15•, 29].

In the general population, there is a gap in the age of onset of ASCVD with the first ASCVD event occurring 7–10 years later in women than in men [13, 14]. In the FH population, the first ASCVD event is observed to occur at a similar age in women and men with FH [11, 41]. In a Norwegian cohort, mean age at first ASCVD event was 46 years in women and 43 years in men [11]. This contrasts with the age gap in the general population. However, some studies report an age gap in ASCVD onset also in FH [15•, 43].

The excess ASCVD risk in FH seems to be higher in female FH patients compared to male FH patients [15•, 16, 41]. Especially in the youngest age groups, an increased risk of acute myocardial infarction (MI) has been observed, with a standardized incidence ratio of 13.6 and 7.5 for women and men with FH (25–39 years), respectively, in a Norwegian study [16]. Excess CVD morbidity in young women (30–50 years) with FH was also observed in the UK Simon Broome register [15•]. However, others describe an equal excess ASCVD risk in women and men [30].

With regard to HoFH, no differences were observed in age at first ASCVD event between women (median 30 [17–40] years) and men (median 28 [17–40] years) in the previously mentioned global HoFH registry, suggesting a larger impact of HoFH on women compared to men [28].

## Family Planning and Pregnancy

### Loss of Treatment Periods in Childbearing Age

In female FH patients, treatment periods are interrupted during childbearing age. Lipid-lowering medication, including statins, ezetimibe, and PCSK9-inhibitors, are contraindicated during conception, pregnancy, and breastfeeding, although the level of evidence is low (class III level C) [7]. Bile acid sequestrants that are not absorbed and/or LDL apheresis may be considered for women with severe FH [7]. When a woman with FH is planning pregnancy, she must discontinue statin treatment, resulting in a rapid increase of her LDL-C level to pre-treatment levels. When she becomes pregnant, the LDL-C level increases even further due to the normal physiological changes during pregnancy. The increase in LDL-C levels among women with FH is similar as in women without FH (approximately 30 % both in women with and without FH) [44]. However, in women with FH, the absolute LDL-C level is much higher, reaching a mean LDL-C level above 8 mmol/L in gestational week 30 [44], and clinical examples show LDL-C levels up to 12 mmol/L in gestational week 36 (Clinical Trials: NCT05367310).

We previously observed that women with FH experience a median loss of 2.3 years of treatment time due to family planning. However, there was a considerable degree of individual variation, ranging from 0 to 14 years off treatment due to family planning [45•], and 20 % of the women had more than 4 years lost treatment time. A recent Australian case series on 13 women with FH found a similar mean 2.3 years of treatment loss from planning of pregnancy to the end of breastfeeding [46]. We also observed that the pregnancy-related off-treatment periods constituted of approximately 20 % lost treatment time at the mean age of 31 years at last pregnancy in women with FH in Norway and the Netherlands [45•].

Clinical examples show that the off-treatment period can be extended between multiple pregnancies, and 10 years off-treatment has been observed from planning of the first pregnancy to end of breastfeeding of the third child, where statin treatment was not restarted in between pregnancies. A 10-year period or longer pregnancy-related off-treatment time will therefore contribute to increased LDL-C burden in women with FH. To our knowledge, no studies have been conducted to investigate whether these off-treatment periods impact the risk of ASCVD later in life and whether this may contribute to the disappearance of the 7- to 10-year age gap between women and men normally seen in the general population.

### Breastfeeding

There are no specific recommendations for breastfeeding in FH, and women with FH are recommended to breastfeed in line with the general population. The World Health Organization recommends exclusive breastfeeding for the first 6 months and then continued partial breastfeeding for up to 2 years of age or longer [47]. We previously showed that women with FH breastfed to a lesser extent than the general population, both in Norway and the Netherlands. The duration of breastfeeding was also shorter than in the general population in the respective countries, with a median 8.5 and 3.6 months in women with FH in Norway and the Netherlands, respectively [45•].

To our knowledge, there are no studies on the effects of breastfeeding in women with FH. Data from the general population have shown that breastfeeding has several benefits both for the mother and child. For the child, breast milk prevents infections by providing antibodies for the child [48, 49]. For the mother, breastfeeding can promote postpartum weight loss, can improve lipid profile, and is important for mother-child bonding [48, 49]. There is also some evidence that breastfeeding can reduce the risk of type 2 diabetes and CVD [48].

### Management of FH in Childbearing Age

Early initiation of treatment is of high importance in female FH patients to compensate for pregnancy-related off-treatment periods later in life. Focusing on lowering LDL-C levels before, in between, and after childbearing age can compensate for the increase in the lifelong cholesterol burden caused by off-treatment periods. This is currently not addressed in the European clinical guidelines on the management of dyslipidemias and CVD prevention [7]. In 2011, the American Heart Association presented women-specific guidelines for CVD prevention [50]. These guidelines take into account sex-specific risk factors for CVD (e.g., the hormonal changes during pregnancy and menopause) and were a milestone to ensure increased knowledge and proper treatment for women. However, no new women-specific guidelines have been published since. It is also noteworthy that in these guidelines, specific recommendations for women with FH were not addressed.

### Family Planning Periods: a Patient Perspective

In our pilot study in the Netherlands and Norway, we found that only a minority (13–29 %) of women with FH had an appointment with a physician specialized in FH during their first pregnancy [45•]. Additional follow-up by a FH specialist during pregnancy is usually not part of the standard treatment; however, most women (86 %) indicated a need for more information regarding pregnancy and breastfeeding related to having FH, and almost half (47 %) reported concerns related to medication use during pregnancy. It is unknown how men with FH experience family planning nor is it known how FH healthcare professionals provide information regarding family planning to patients with FH.

In accordance with the new International Atherosclerosis Society (IAS) guidance for best practice of FH care, both female and male patients should receive counselling on family planning early on and women with an active pregnancy wish should be referred to a dedicated FH center to make an individualized family planning plan [51••].

The majority of patients with HoFH have distress about having children, and women with HoFH have even more concerns compared to men due to the impact of treatment interruptions [52–54]. The 2023 EAS HoFH consensus statement addresses this issue and recommends individualized care in all stages of family planning [6].

## Future Directions

**Fig. 1:**
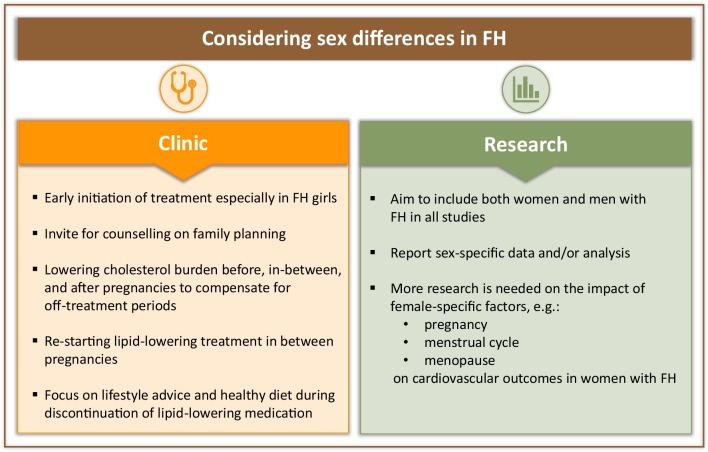
Future directions for considering sex differences in FH in clinic and research. FH: familial hypercholesterolemia

Future directions for considering sex differences in FH in clinic and research are summarized in Fig. 1.

### In Clinic

In clinical practice, it is important that both female and male patients with FH are diagnosed earlier in life and at a similar age to initiate treatment as early as possible. Especially in girls and young women with FH, treatment should be further optimized in view of the higher LDL-C burden encountered already early in life.

Treatment of FH patients should take place in dedicated FH centers. Both women and men with FH of reproductive age should be counselled about family planning.

Clinical guidelines for treatment of FH should focus on the importance of lowering the LDL-C burden in female FH patients before, in between, and after pregnancies to compensate for pregnancy-related off-treatment periods. Re-starting lipid-lowering treatment in between pregnancies, even if only for a few months, can contribute to lowering the lifelong cholesterol burden. This should also be incorporated in guidelines for general physicians, who might be the primary healthcare contact for women during pregnancy periods.

In periods of discontinuation of lipid-lowering treatment during pregnancy and breastfeeding, focus on lifestyle advice and a healthy diet for mother and child should be emphasized. Women should be advised of the importance of restarting lipid-lowering medication promptly after breastfeeding.

### In Research

More research should be performed regarding patient outcomes in FH, such as family planning, in both women and men. Future research should aim to include both sexes and an equal proportion of women and men in clinical trials and include populations from different geographic regions. In addition, sex-specific data and/or analysis should always be reported regardless of the outcome.

Furthermore, future research on the effect of female-specific life stages, including menstrual cycle, pregnancy, breastfeeding, and menopause on lipid levels, is needed and should investigate whether these life stages impact the future risk of ASCVD in women with FH and HoFH.

Ultimately, sex-specific FH research may translate into sex-specific clinical guidelines to improve the care currently given.

## Conclusions

Several studies show that FH impacts women through life differently than men in terms of women having higher untreated and treated LDL-C levels, less intensive treatment, off-treatment periods due to family planning, and a possibly excessive ASCVD impact compared to men. Earlier initiation and lifelong treatment, especially in young women with FH, are important to lower the lifelong cholesterol burden. Future research on the impact of female-specific life stages such as pregnancy, breastfeeding, menstrual cycle, and menopause is needed. Future guidelines should include sex-specific aspects.
